# Trematocine, a Novel Antimicrobial Peptide from the Antarctic Fish *Trematomus bernacchii*: Identification and Biological Activity

**DOI:** 10.3390/antibiotics9020066

**Published:** 2020-02-06

**Authors:** Giulia Della Pelle, Giulia Perà, Maria Cristina Belardinelli, Marco Gerdol, Martina Felli, Silvia Crognale, Giuseppe Scapigliati, Francesca Ceccacci, Francesco Buonocore, Fernando Porcelli

**Affiliations:** 1 Department for Innovation in Biological, Agrofood and Forest Systems, University of Tuscia, 01100 Viterbo, Italy; g.dellapelle@unitus.it (G.D.P.); giulia.p193@gmail.com (G.P.); belardinelli@unitus.it (M.C.B.); martinafelli@live.it (M.F.); crognale@unitus.it (S.C.); scapigg@unitus.it (G.S.); porcelli@unitus.it (F.P.); 2 Department of Life Sciences, University of Trieste, Trieste 34128, Italy; marco.gerdol@gmail.com; 3 CNR—Institute for Biological Systems, Sede Secondaria di Roma-Meccanismi di Reazione, 00185 Rome, Italy; francesca.ceccacci@cnr.it

**Keywords:** antimicrobial peptides, model membranes, fish immune system, Antarctica

## Abstract

Antimicrobial peptides (AMPs) are short peptides active against a wide range of pathogens and, therefore, they are considered a useful alternative to conventional antibiotics. We have identified a new AMP in a transcriptome derived from the Antarctic fish *Trematomus bernacchii*. This peptide, named Trematocine, has been investigated for its expression both at the basal level and after in vivo immunization with an endemic Antarctic bacterium (*Psychrobacter* sp. TAD1). Results agree with the expected behavior of a fish innate immune component, therefore we decided to synthesize the putative mature sequence of Trematocine to determine the structure, the interaction with biological membranes, and the biological activity. We showed that Trematocine folds into a α-helical structure in the presence of both zwitterionic and anionic charged vesicles. We demonstrated that Trematocine has a highly specific interaction with anionic charged vesicles and that it can kill Gram-negative bacteria, possibly via a carpet like mechanism. Moreover, Trematocine showed minimum inhibitory concentration (MIC) and minimum bactericidal concentration (MBC) values against selected Gram-positive and Gram-negative bacteria similar to other AMPs isolated from Antarctic fishes. The peptide is a possible candidate for a new drug as it does not show any haemolytic or cytotoxic activity against mammalian cells at the concentration needed to kill the tested bacteria.

## 1. Introduction

Due to the dramatic increase of conventional antibiotic resistant bacterial strains, the need for new antimicrobial molecules is ever-increasing. Antimicrobial peptides [1] are a ubiquitous class of small proteins regarded as the most promising candidate to fight against antibiotic resistance. Although the presence of antibiotic peptides in bacteria has been known since gramicidine A and B were isolated in the late 1930s, the first established antimicrobial peptide from animals was mellitin, an AMP from *Apis mellifera* venom [2,3]. Since then, AMPs have been found in every life kingdom, also in Protista [4], Plants [5,6], and Fungi [2,7,8]. It was discovered, throughout the years, that AMPs represent a very ancient class of innate immunity effectors [9,10,11], despite their wide diversity in terms of structure, biosynthesis, physico-chemical characteristics, and length [12,13]. Usually, they are mainly classified by taking into consideration their structure (α-helices, β-sheets, extended or loop structures). Moreover, α-helix AMPs usually show an amphipathic structure [14,15,16]. Their main target is the plasma membrane [1], and so far it has been proved that their selectivity relies on both their sequence and membrane charge density [14,15,17,18] because bacterial cell walls show, usually, an anionic charge. AMPs are mostly able to partition into membrane bilayers, form pores, or cause perturbation of a different nature into the bilayer [19]. Despite being a promising alternative to traditional antibiotics, only a few antimicrobial peptides are currently in clinical trials [12,20]; this is due mainly to their not yet competitive costs and some regulatory issues. They have also been investigated as anti-tumor agents [21], but their best application seems to be as antimicrobial agents against multi-drug resistant microbes [15,20,22,23].

Antarctica marine life is an excellent source for novel drugs due to its peculiar environment and to the specific adaptations that the species living there have evolved. Antarctic Notothenioidei have been shown to express some piscidins [24,25], a promising class of AMP, structurally related to the well-known cecropins from insects [2]. We previously isolated a piscidin peptide from the icefish *Chionodraco hamatus*, named Chionodracine [26], and we developed a number of mutants from its sequence [15,27], which have proven to be able to effectively kill multi-drug resistant bacteria. Here, we present the isolation, membrane interaction, and antimicrobial activity of a new peptide from a red-blooded Antarctic fish, *Trematomus bernacchii* that we have named Trematocine.

## 2. Results

### 2.1. Identification of Trematocine: A Piscidin

**Figure 1:**
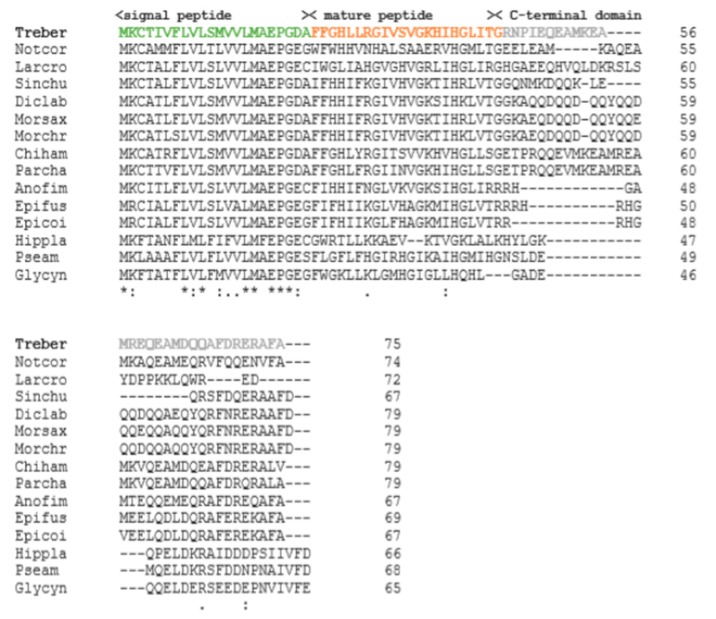
Multiple alignment of the predicted Trematocine amino acid sequence with other known AMP molecules from Teleost fish (accession numbers: *Trematomus bernacchii*, MH325166; *Notothenia coriceps*, XP_010772966; *Larimichthys crocea*, EU741827; *Siniperca chuatsi*, AAV65044; *Dicentrarchus labrax,* AAP58960; *Morone saxatilis*, AF385583; *Morone chrysops*, AAL57318; *Chionodraco hamatus*, FR718953; *Parachaenichthys charcoti*, AOW44479; *Anoplopoma fimbria*, ACQ58110; *Epinephelus fuscoguttatus*, ADE06665; *Epinephelus coioides*, AY705494; *Hippoglossoides platessoides*, AY273174; *Pseudopleuronectes americanus*, AY282498; and *Glyptocephalus cynoglossus*, AY273177).

The nucleotide sequence of the antimicrobial peptide Trematocine, found in the head kidney transcriptome, was confirmed by cDNA cloning with primers that amplify its entire coding region (data not shown). This sequence encodes for a pre-pro-peptide of 75 amino-acids. A multiple alignment of the amino-acid sequence of the Trematocine with other known AMP sequences from Teleosts was assembled to investigate the conserved amino acid residues (Figure 1). From the alignment, it can be evidenced that only a few amino-acids are conserved between the various species, and these amino-acids are mainly located in the N-terminal region. Moreover, with the Signal p 4.0 program, it was possible to identify the region of the signal peptide that falls between position 1 and 22. Comparing the pro-peptide of the Trematocine to the mature peptides already identified in other Teleosts, we obtained the putative sequence of the biologically active molecule (mature peptide).

### 2.2. Basal and Stimulated Expression in Different Tissues

**Figure 2:**
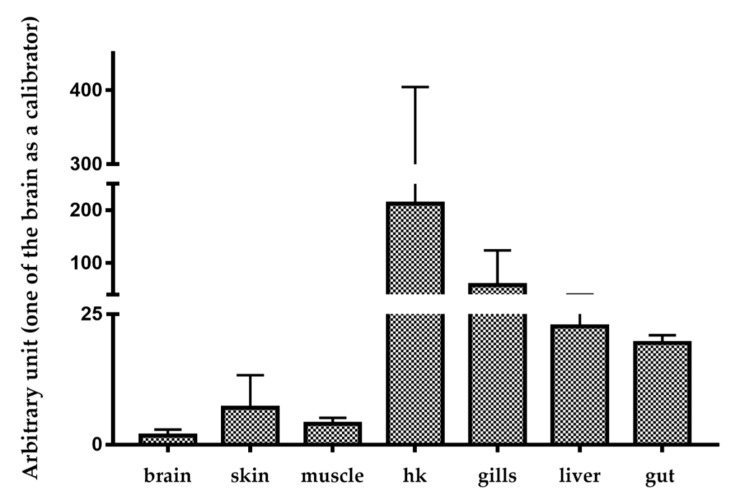
Trematocine basal expression in different tissues. Trematocine mRNA levels were expressed as a ratio to 18 S rRNA levels in the same samples after real-time PCR analysis using the tissue with the lowest expression (brain) as the calibrator.

**Figure 3:**
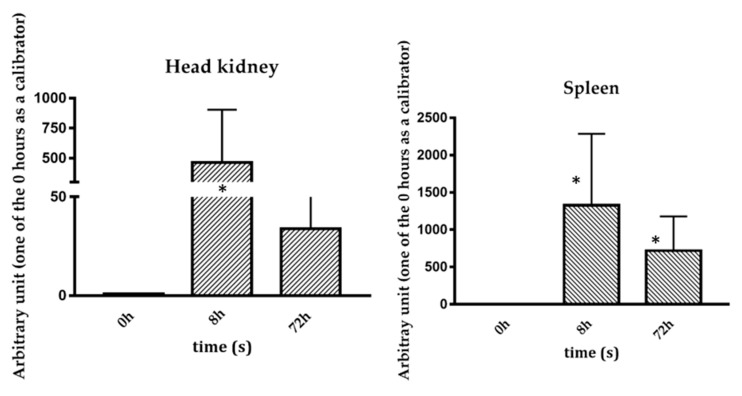
Trematocine expression analysis after in vivo immunization with *Psychrobacter* sp. TAD1 in head kidney and spleen after 8 h and 72 h. The results are expressed as mean + SD and the asterisk indicate the significance level with respect to control (0 h). * = *p* < 0.05.

Trematocine mRNA basal levels have been analyzed in different organs and tissues of *T. bernacchii* healthy animals. The highest Trematocine expression was found in head kidney (HK) followed closely by the gills. The lowest mRNA level was found in the brain (Figure 2). The constitutive expression in organs and tissues fundamental for fish immune responses is high and, therefore, indicative of a molecule possibly involved in the innate immune system. Trematocine expression after in vivo stimulation with *Psychrobacter* sp. TAD1 (Figure 3), an endemic Antarctica bacterium [1], shows a significant and relevant increase after 8 h in both head kidney and spleen leukocytes.

### 2.3. Trematocine Mature Peptide Structure

**Figure 4:**
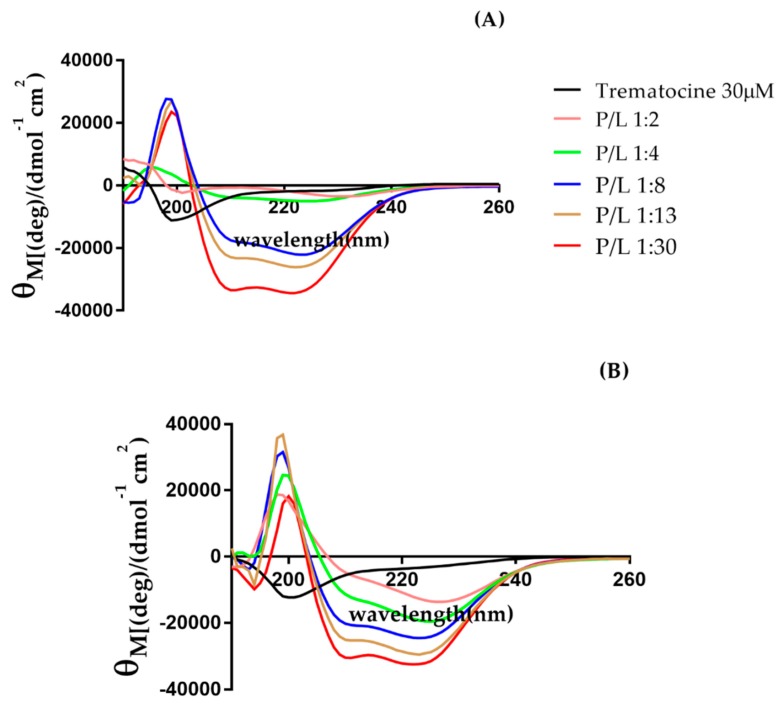
Circular dichroism spectra for Trematocine upon addition (**A**) 1-palmitoyl-2-oleoyl-sn-glycero-3-phosphoethanolamine (PE)/1-palmitoyl-2-oleoyl-sn-glycero-3-phosphoglycerol (PG) (3:1) large unilamellar vesicles (LUVs), and (**B**) 100% 1-palmitoyl-2-oleoyl-glycero-3-phosphocholine (PC) LUVs. P/L ratios are expressed in mol/mol, and the raw signal is corrected in Ѳ_M_.

**Figure 5:**
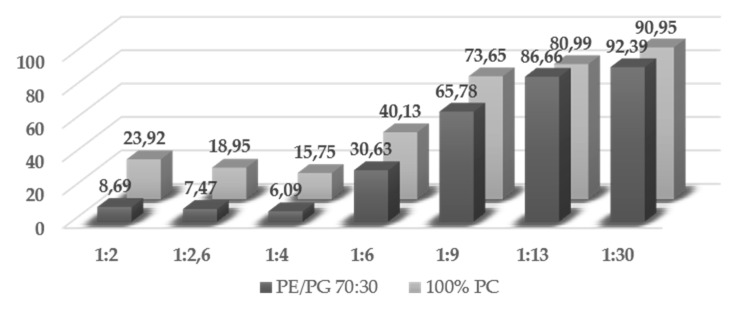
α-helix percentages obtained via the K2D3 algorithm using spectra obtained as explained in Section 4.5. A slightly higher helicity in the presence of PE/PG 70:30 LUVs can be noted.

Evaluation of the Trematocine secondary structure was carried out by circular dichroism spectroscopy, as described in Section 4.3. In buffer solution, the peptide was unstructured, as shown by the minimum found around 200 nm. Upon addition of increasing amounts of large unilamellar vesicles (LUVs), it gradually switched to a α-helix configuration, as revealed by the two minima at 208 nm and 222 nm (Figure 4). This happened in the presence of both anionic (PE) and zwitterionic lipid LUVs, with a significant switch at the 1:9 P/L ratio. Interestingly, there was a faint 230 nm minimum at the 1:2 P/L ratio, peculiar of interactions among aromatic residues [28], putatively the N-terminal Trp and Phe, thus suggesting a cooperative effect among peptide monomers. The results are summarized in Figure 5, with the relative helical percentages (obtained via the K2D3 [29] algorithm) reported in both for zwitterionic and anionic LUVs. A slightly higher helicity is obtained in the presence of PC/PG 70:30 LUVs.

### 2.4. Trematocine Model Membranes Interaction

**Figure 6:**
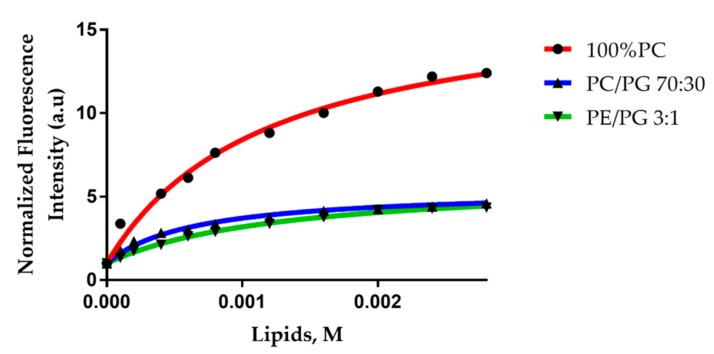
Binding isotherms for Trematocine interacting with LUVs of different compositions. Tryptophan fluorescence was measured. Trematocine concentration was 1 µM.

LUVs are considered a reliable model to study the interaction between a peptide and a double layer. In order to have a suitable curvature radius, we chose to use LUVs with an average diameter of 100 nm. We studied the interaction by steady state fluorescence spectroscopy, following the emission of Trp. To evaluate the interaction between Trematocine and different LUVs, we detected the Trp-1 fluorescence upon partitioning in the presence of LUVs. The Trp emission showed a blue shift and an increase of intensity, typical of a transition from a polar to a non-polar environment. Binding isotherms are reported in Figure 6, and in Table 1 are shown the mole fraction partition constants (1) [27,30].

(1) $$f_{p}=\frac{K_{x}\left[L\right]}{K_{x}\left[L\right]+\left[W\right]}$$

These results evidenced that Trematocine interacts with model membranes, with a preference (*K_x_* = 8.8 × 10^4^) for PC/PG anionic (70:30) LUVs, the most used model for bacterial membranes, while the interaction with PE/PG 3:1 and 100% 1-palmitoyl-2-oleoyl-glycero-3-phosphocholine (PC) LUVs is weaker (*K_x_* ~ 4 × 10^4^). This demonstrates a distinct selectivity towards negatively-charged membranes. Furthermore, to evaluate the topology of the peptide interacting with different LUVs, we studied the quenching of Trp fluorescence by acrylamide. As expected, the Stern–Volmer constants (Table 2) were higher in the buffer (21.2 M^−1^) than in the presence of vesicles (~1 M^−1^). This suggests that the N-terminal Trp of Trematocine is accessible in the buffer while it partitions and inserts in the interfacial region of the double layer in presence of LUVs. We also calculated the Net Accessibility Factor (NAF) [31], defined by Equation (2).

(2) $$NAF=\frac{K_{SV\;\left(LUV\right)}}{K_{SV\;\left(BUFFER\right)}}.$$

**Figure 7:**
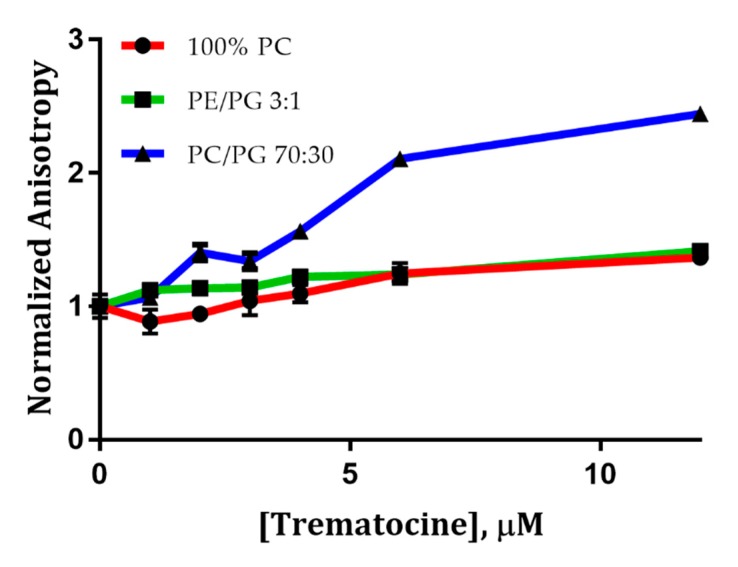
Steady-state fluorescence anisotropy relative to 1,6-Diphenyl-1,3,5-hexatriene (DPH)-containing LUVs, upon interaction with Trematocine. Anisotropy was normalized for each experiment with the r_0_ relative to DPH-containing LUVs in the absence of the peptide.

NAF values, reported in Table 2, were lower in the presence of PE/PG 3:1 LUVs, suggesting a lower accessibility of Trp-1 to the quencher in the presence of phosphatidiletanolammine head groups [32] rather than phosphatidilcoline ones. We then carried out studies of fluorescence anisotropy using the 1,6-Diphenyl-1,3,5-hexatriene (DPH) fluorescent probe. DPH is a compound whose fluorescence anisotropy depends on the membrane fluidity. In Figure 7, the normalized anisotropy of DPH, measured at 25 °C, is reported at different concentrations of peptide. Only for PC/PG LUVs does the anisotropy of DPH vary, indicating a decrease of the membrane fluidity upon addition of peptide (Figure 7). For PC and PE/PG LUVs, the anisotropy is essentially the same, indicating that membrane fluidity is not affected by the addiction of peptide. These studies confirmed the same trend we reported in the partition experiments, showing, a greater interaction between the Trematocine and PC/PG (70:30) double layer rather than 100% PC and PE/PG (3:1) LUVs.

### 2.5. Trematocine Crosses Outer Membrane and Interacts with Inner Membrane

**Figure 8:**
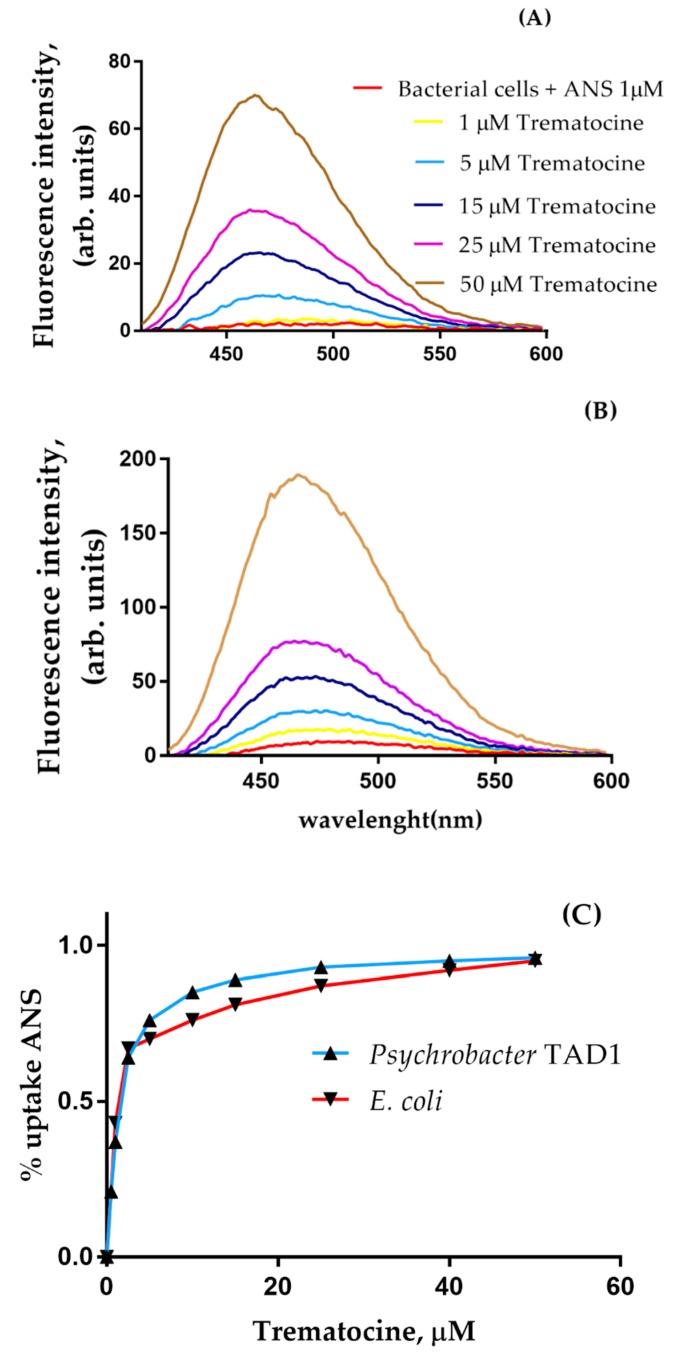
Permeabilization of *Psychrobacter* TAD1 (**A**, **B**) *E. coli*, outer membrane by Trematocine. (**C**): percentage of ANS uptake.

**Figure 9:**
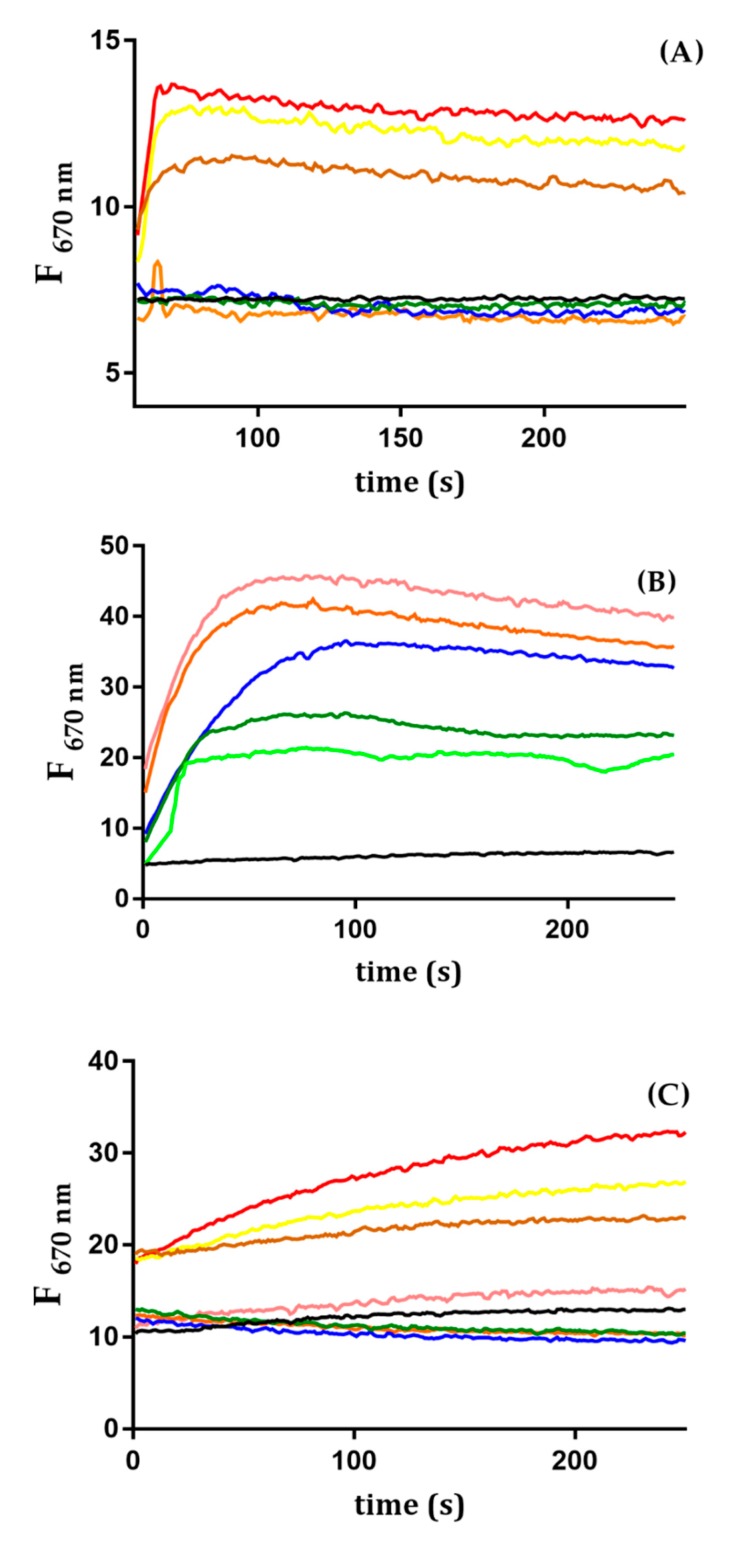
Inner Membrane depolarization measured using Disc3(5): (**A**) *Psychrobacter* TAD1, (**B**) *B. pumilus*, (**C**) *E. coli* at different Trematocine concentrations. For every species, the baseline is in black, then: light green, 10 nM; dark green, 20 nM; blue, 50 nM; orange, 100 nM; pink, 250 nM; brown, 0.5 µM; yellow, 1 µM; and red, 2 µM.

In order to prove the ability of Trematocine to disrupt the Gram-negative bacteria outer membrane (OM), an ANS assay was performed. As shown in Figure 8, for both *E. coli* and *Psychrobacter* sp. TAD1, the addition of Trematocine causes a sharp increase of ANS fluorescence, in a dose-dependent way. This is due to the capability of the probe to translocate inside the periplasmatic space because of a perturbation in the lipopolysaccharidic wall. Furthermore, inner membrane (IM) depolarization assay, using a Disc3(5) fluorescent probe, showed that Trematocine can disrupt the electric gradient of *E. coli*, *Psychrobacter* sp. TAD1, and *Bacillus pumilus*, a Gram-positive bacteria cell walls in a dose-dependent way (Figure 9), proving again that Trematocine can interfere with the integrity of the anionic-charged phospholipidic double-layer. The effect was more evident on *B. pumilus*, with a detectable depolarization at a concentration of Trematocine 10 nM. Interestingly, for *E. coli*, an initial hyperpolarization (indicated by the decrease of Disc3(5) fluorescence) has been observed at low peptide concentrations, hinting at the need for a possible threshold concentration to disrupt membrane potential.

### 2.6. Antimicrobial Activity

The Trematocine concentrations required to inhibit and kill the tested bacterial strains are summarized in Table 3. Trematocine shows activity against both Gram-positive and Gram-negative bacteria. Among all tested bacterial strains, *Psychrobacter* sp. TAD1 and *Bacillus pumilus* were the most susceptible. A certain antimicrobial activity was also registered toward yeast cells.

### 2.7. Haemolytic and Cytotoxic Activity

**Figure 10:**
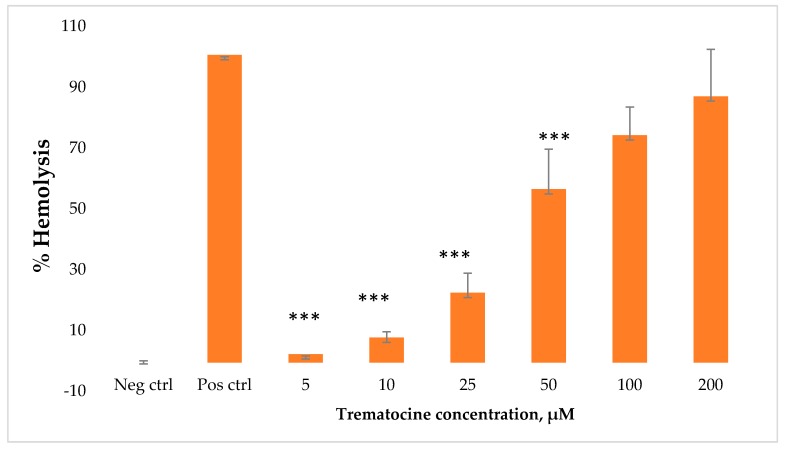
Hemolytic activity of Trematocine against rabbit erythrocytes. Six different concentrations have been tested. The values represent the mean + SD, and the asterisks indicate the significance level with respect to positive control (100% haemolysis): *p* < 0.01 ***.

The hemolytic effect of Trematocine has been tested on rabbit erythrocytes to investigate its capacity to induce membrane lysis. Six different concentrations of peptide have been tested (from 5 μM to 200 µM). Hemolysis rates at the lowest concentrations (5 μM and 10 µM) are between 1% and 7%. A notable increase of hemolytic effect can be seen above a concentration of 50 μM, with a 55% hemolysis (Figure 10).

**Figure 11:**
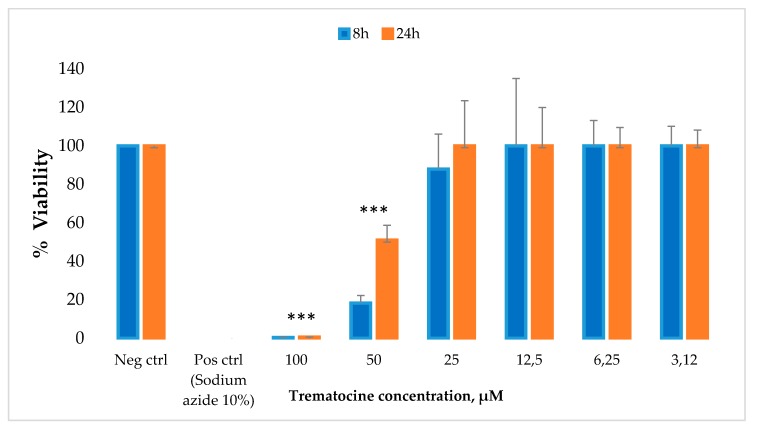
Cytotoxic activity of Trematocine against a primary human fibroblast cell line (FB789). Six different concentrations have been tested. The values represent the mean + SD, and the asterisks indicate the significance level with respect to negative control (100% of viability). *** = *p* < 0.001.

We have also studied the effect of Trematocine peptide on a primary human fibroblast cell line (FB789). The peptide was tested at six concentrations, and the percentage of cell viability was evaluated after 8 h and 24 h. The results (Figure 11) showed that Trematocine is toxic at very high concentrations (100 µM and 50 μM), while at lower concentrations (from 25 µM to 3.12 μM) it did not show any toxicity.

## 3. Discussion

The current emergence of pathogen antibiotic resistance has given a great boost to the search for novel drugs. Antimicrobial peptides [1] are a widespread and differentiated class of molecules, mainly with antibiotic (and sometimes antifungal and antiviral [33]) properties. Since their discovery in the late 1970s, more than 5000 molecules have been characterized. The sea environment is a rich source of such entities, due to its peculiar microbial promiscuity. In particular, Antarctica has been selected as an excellent field of discovery for new AMPs with specific structures due to its peculiar environmental conditions [25,26,27]. From the analysis of a *Trematomus bernacchii* transcriptome [34] we identified a putative sequence of an AMP, included in a family of piscidins that we named Trematocine. It shows a remarkable homology to another AMP we identified from an icefish, the Chionodracine, and lower identity with other AMP from Teleost fish, such as Dicentracine from *Dicentarchus labrax* [35]. Usually, in mammals, AMPs are produced as pre-pro-peptides and, after secretion, the mature peptide is obtained due to the processing of the pro-peptide by proteases [36]. The only region that is highly conserved in all peptides is the signal peptide and, therefore, it could be argued that they all use the same secretion pathway. To verify that this sequence really corresponds to an AMP involved in the *T. bernacchii* innate immune responses, we investigated its basal expression in different tissues and organs. The highest expression was found in head kidney and the gills; the first is known as the main lymphoid tissue in teleost fishes [33,37,38], whereas the second is the main pathogen portal of entry. High levels of expression in head kidney (HK) have been found for other fish AMPs, like for Dicentracine in sea bass [38] and Epinecidin in orange-spotted grouper [39]. Trematocine expression in *T. bernacchii* skin—and so, its presence in mucus—is very low compared to other AMP frequently found in fish mucus [40,41,42,43]. Thus, probably, Trematocine is mainly a leucocyte effector rather than a first barrier AMP. This could explain its very low cytotoxicity, comparable to other native AMPs with similar expression patterns [26,27]. Furthermore, we attempted to elicit its expression upon stimulation through in vivo immunization with *Psychrobacter* sp. TAD1, an endemic bacterial pathogen of Antarctica. We showed that the AMP is up-regulated in HK and spleen, as foreseen. In gilt-head seabream, a similar expression pattern was found for a hepcidine [44], and, in *Oryza melastigma*, upon stimulation with *Vibrio parahaemolyticus*, a known Gram-negative pathogen bacteria, despite *O. melastigma* being evolutionary distant from *T. bernacchii*. Among piscidins [24,45], GAD-1 and GAD-2 transcripts were up-regulated only in the spleen through the injection of bacterial antigens (ASAL) in *Gadus morhua* [46]. Given this data, expression in tissue different from gills, spleen, and of course HK, can be explained with the presence of circulating Trematocine—expressing mast cells due to their central role in innate immunity of teleost fishes [47]. Due to these encouraging preliminary results, we decided to investigate the structure of the Trematocine putative mature peptide with the aim of verifying its resemblance to other piscidins. Through circular dichroism spectroscopy, we proved that—as with most helical AMPs—Trematocine is a random coil in the buffer, but it develops a consistent alpha helix structure in the presence of lipid vesicles, both zwitterionicly and anionicly charged. As is peculiar with many AMP families, Trematocine α-helix has an amphipatic structure. Trematocine, thus, also exhibits a higher thermodynamic affinity, as it can be inferred by the Wimley [30] partition constants, for anionic charged double layers rather than zwitterionic; this leads to a good selectivity ratio defined by the ratio between the partition constants measured in PC/PG, PE/PG, and POPC [48,49]. Furthermore, as evidenced by Trp quenching analysis, Trematocine deeply inserts into the double layer with a slight preference for 1-palmitoyl-2-oleoyl-sn-glycero-3-phosphoglycerol (PG) containing LUVs, a common feature for cationic charged AMPs [50,51,52,53,54]. Also, fluorescence anisotropy gave some interesting clues about the Trematocine mode of action towards lipid membranes. DPH fluorescence is related to membrane microviscosity [55]; pore-forming drugs and cholesterol usually cause a decrease in membrane anisotropy due to an augmented packing of acyl tails [56,57,58,59,60]. Interestingly, upon addition of Trematocine, DPH fluorescence anisotropy in anionic LUVs increased, thus indicating a different mechanism in the perturbation of the double layer, decreasing its fluidity, in a similar fashion of a bacteriocin, the famous magainin 2 and mellitin [41,61,62], rather than other elicoidal AMPs. All these data, taken together, confirm the hypothesis that Trematocine follows a carpet like mode of action [14,63], as it has been demonstrated for the related Chionodracine [15,26]. Further studies (i.e., electronic microscopy and molecular dynamics simulations) are needed to completely elucidate its mechanism of action. However, we decided to explore its antimicrobial activity against bacteria and yeast. Similar Chionodracine behavior has also been observed in term of antimicrobial activity, being Trematocine active toward both Gram-positive and Gram-negative bacteria, with minimum inhibitory concentration (MIC) values around 2.5–25 μM, especially against Gram-negative bacteria. Trematocine has also been shown to possess anti-fungal activity against *Candida boidinii* [64], demonstrating that the peptide, acting on membranes, possessed broad-spectrum antimicrobial activity. To investigate its potential as a new candidate drug we determined both the haemolytic activity against mammalian erythrocytes and the cytotoxicity against a human primary cell line. At the peptide concentration useful to kill the tested bacteria, there is no evidence of any Trematocine negative effect.

## 4. Materials and Methods

### 4.1. Identification of an AMP from T. bernacchii

The nucleotide sequence of a putative antimicrobial peptide was identified from the head kidney transcriptome of *T. bernacchii* [34]. Adult specimens of *Trematomus bernacchii* were collected in various Antarctic campaigns by researchers of the Italian National Research Project in Antarctica (PNRA) at the Italian Antarctic Base, Terra Nova Bay, Ross Sea (Mario Zucchelli Station). After collection, fish were placed in tanks with running seawater. Organs and tissues (brain, skin, muscle, head kidney, gills, liver, and gut) from *T. bernacchii* were sampled and homogenized, disrupting them by teasing on a 100 mm cell strainer; the obtained cells were placed in eppendorfs containing Tripure (Roche, Basel, Switzerland) for total RNA extraction. The total RNA was resuspended in DEPC-treated water. Controls for the quality of cDNA were performed using actin primers that spam an intron (see Table 4). PCR reactions were conducted using a Mastercycler (Eppendorf, Hamburg, Germany). The cycling protocol was one cycle of 94 °C for 5 min, 35 cycles of 94 °C for 45 s, 52 °C for 45 s, and 72 °C for 45 s, followed by one cycle of 72 °C for 10 min. PCR products were visualized on 1.5% (*w*/*v*) agarose gels containing Gel red (Biotium, Fremont, CA, USA) and using hyperladder IV (Bioline, Memphis, TN, USA) as size markers. Further primers (see Table 4) were used to amplify the complete sequence of the Trematocine gene based on the sequence found in the transcriptome using as a template a cDNA from head kidney. The PCR product was purified using a QIAquick Gel Extraction Kit (QIAgen, Hilden, Germany), and it was inserted into the pGEM-T Easy vector (Promega, Madison, WI, USA) and transformed into competent JM 109 *Escherichia coli* cells. Plasmid DNA was purified using the Wizard Plus SV Minipreps DNA purification System (Promega) and sequenced by Eurofins Genomics (Ebersberg, Germany). The AMP sequence was analyzed for the presence of a signal peptide using SignalP software [65].

A multiple alignment with other AMP sequences from fish was generated using the CLUSTAL ω program. 

### 4.2. Basal Expression of Trematocine 

To investigate the basal expression of Trematocine, three fish were sampled, and some tissues (brain, skin, muscle, head kidney (HK), gills, liver, and gut) were obtained as described above. Total RNA was isolated from each tissue with Tripure (Roche), resuspended in DEPC-treated water, and used for real-time quantitative PCR. The expression level of Chionodracine transcripts was determined with an Mx3000P real-time PCR system (Stratagene, San Diego, CA, USA) equipped with version 4.1 software and using the Brilliant SYBR Green Q-PCR Master Mix (Agilent Technologies, Santa Clara, CA, USA), following the manufacturer’s instructions, with ROX as an internal passive reference dye. The reaction was performed using primers for the amplification of about 120 bp of the product from Trematocine and 18 S ribosomal RNA used as a house-keeping gene (see Table 4). The PCR conditions were: 95° for 10 min, followed by 35 cycles of 95 °C for 45 s, 52 °C for 45 s, and 72 °C for 45 s. Duplicate reactions were performed for each template cDNA. A relative quantitation was performed, comparing the levels of the target transcript (Trematocine) to a reference transcript (calibrator, the tissue with the lowest Trematocine expression), in this case, the brain. 

### 4.3. Expression after In Vivo Immunization with Psychrobacter sp. TAD1

The in vivo Trematocine expression was studied using total RNA isolated from head kidney (HK) and spleen from three fish stimulated with *Psychrobacter* sp. TAD1. 50 μL of a solution with *Psychrobacter* sp. TAD1 adjusted to 10^9^ cells/mL, together with 50 μL of Freund’s incomplete Adjiuvant, was intraperitoneally injected in fish in Antarctica. After immunization, the fish were placed in aerated tanks until sampling of the selected tissues took place (8 h and 72 h). The primers and the real-time PCR conditions were the same as described in the Section 4.2. The calibrator was the time 0 control. The results of all the experiments were expressed as the mean + SD of the results obtained from three fish at each sampled time, and the differences from the control were considered significant if *p* < 0.05 using t two-way ANOVA analysis followed by the Bonferroni’s post-test. Duplicate reactions were performed for each template cDNA.

### 4.4. Peptides and LUVs Preparation

The peptides (98% purity) were purchased from CASLO ApS (Kongens Lyngby, Denmark). Peptide concentration was determined for each sample preparation by UV light absorption at 280 nm. Large unilamellar vesicles (LUVs) composed, respectively, of 100% 1-palmitoyl-2-oleoyl-sn-glycero-3-phosphocholine (PC), 70%/30% (*w*/*w*) POPC/1-palmitoyl-2-oleoyl-sn-glycero-3-phosphoglycerol (PG), and 3:1 *m*/*m* 1-palmitoyl-2-oleoyl-sn-glycero-3-phosphoethanolamine (PE)/PG were prepared according to the general procedures previously reported [15]. Briefly, the lipids, dissolved in chloroform/methanol, 9:1, were dried under rotary evaporation and then overnight under high vacuum. The lipid film was then hydrated in 1 mL of buffer (20 mM phosphate buffer at pH 7.4 with 150 mM NaCl and 0.8 mM EDTA) and subjected to 5 freeze-thaw cycles. The suspension was extruded through a polycarbonate membrane with an Avanti Polar miniextruder (20 times through two-stacked polycarbonate membranes with pore sizes of 100 nm), and the obtained LUVs were used within 48 h of preparation.

### 4.5. Circular Dichroism Studies

The secondary structure of Trematocine (FFGHLLRGIVSVGKHIHGLITG) in the presence of membrane-mimicking systems was evaluated by circular dichroism spectroscopy. All the experiments were carried out on a Jasco spectropolarimeter, with a thermostated cell holder set at 25 °C. The used buffer was 0.01 M PB and 0.08 mM of EDTA, in order to avoid chlorine anion effects on CD spectra. A 30 µM solution of Trematocine was titrated with LUVs of different compositions (100% PC, 70:30 PC/PG, and 3:1 PE/PG). Successively, the resulting spectra were analyzed with the K2D3 algorithm [29]. The reported CD spectra are the average of 16 scans with a scanning speed of 100 nm/min, a response time of 4 s, a bandwidth of 1.0 nm, and a step size of 0.1 nm. The obtained data in millidegrees (mdeg) were converted to mean molar ellipticity per residue (deg cm^2^ dmol^−1^) [66].

### 4.6. Steady-State Fluorescence Studies

All the steady state fluorescence experiments were performed using a Perkin Elmer LS55 operating at 25 °C in a thermostatic cell holder. The spectra were corrected by subtracting the corresponding blanks. Only in this case did we use a mutant of Trematocine (WFGHLLRGIVSVGKHIHGLITG), in which the first Phe was replaced with a Trp, in order to obtain a stronger signal both in UV/Vis and in the fluorescence analysis.

#### 4.6.1. Partition Studies

The ability of peptides to associate with and partition into lipid vesicles was studied by measuring the enhancement of tryptophan fluorescence upon the addition of LUVs. Trp-1 fluorescence spectra were recorded at wavelengths between 310 and 500 nm, considering an excitation wavelength of 295 nm. Measurements were performed with a cross-oriented configuration of polarizers (*Pol_em_* = 90° and *Pol_exc_* = 0°) to reduce contributions from vesicles [67]. A 1.0 mM peptide solution in a 10 mM phosphate buffer at pH 7.4 containing 0.8 mM EDTA and 150 mM NaCl was added to a cuvette and then titrated with LUVs of different compositions (100% PC, 70:30 PC/PG, and 3:1 PE/PG) with a lipid/peptide ratio ranging from 50 to 500, as described previously [26,27]. The background effects of both buffer and vesicles were subtracted from each spectrum. Mole fraction partition coefficients, *Kx*, were obtained, calculating the fraction of peptide, *fp*, which partitioned into the LUVs [30,68,69,70]. The values of *Kx* were obtained, as described before [15,27].

#### 4.6.2. Intrinsic Fluorescence Quenching Studies

Peptide solutions (5.0 mM) in both the absence and presence of LUVs (in a peptide:lipid ratio 1:100), were excited at 295 nm, and fluorescence spectra were recorded from 305 to 500 nm. The samples were titrated by adding increasing amounts of acrilamyde in the range 0.01–0.28 M, and spectra were recorded with excitation and emission band widths of 5 nm. All the fluorescence spectra were corrected for dilution. Fluorescence intensities were extracted, and the data were fitted according to the Stern–Volmer equation, as described previously [26,27,45].

#### 4.6.3. Outer Membrane Permeability Essay

The permeabilization assay was carried out using the fluorescent probe 1-aminonaphtalene-8-sulfonic acid (ANS), as previously described [26,27,71]. *E. coli*, *Psychrobacter* sp. TAD1 [26], and *Bacillus pumilus* strains were grown at 37, 15, and 28 °C, respectively, to mid-log phase in Luria Bertani (LB) broth (Sigma, Darmstadt, Germany); when the corrected growth phase was reached, they were centrifuged at 3000× *g*, washed, and suspended in 10 mM Tris-HCl, 150 mM NaCl, and 0.8 mM EDTA (pH 7.4) buffer, to give an OD_600_ of 0.6. Subsequently, increasing amounts of the Trematocine (from 1.0 to 50 µM) were added to a quartz cuvette containing 1.0 mL of cell suspension and 5.0 µM ANS. Fluorescence spectra were recorded at wavelengths between 400 and 600 nm with an excitation wavelength of 360 nm. The excitation and emission slit widths were 5 nm. After the peptide effect, the ANS was incorporated into the periplasm and, consequently, the fluorescence intensity increased and blue shifted.

#### 4.6.4. Inner Membrane Depolarization Essay

In order to determine if there is any depolarization effect on the above-inidicated bacterial strains, Disc3(5) cyanine dye was used [72]. A cell suspension of each selected bacteria (*E. coli*, *B. pumilus*, *Psychrobacter* TAD1) was incubated with 3 µM Disc3(5) at the right growth temperature, under agitation, for at least half an hour, to allow the probe to insert inside the inner membrane; it was noticed that, according to cell diameter, the optimal OD_600_ was different for each cell type. All the experiments were carried out in the dark in order to avoid photobleaching of the Disc3(5) probe. When a stable decrease in fluorescence was reached, different 1 mL samples were prepared, with Trematocine concentrations ranging from 20 nM to 2 µM. Disc3(5) was excited at 622 nm, and its 670 nm fluorescence emission was recorded in a time-driven experiment. For *E. coli*, the assay was optimized for an OD_600_ of 0.17, for *Bacillus pumilus* of 0.02, and for *Psychrobacter* TAD1 of 0.11.

#### 4.6.5. Fluorescence Anisotropy Studies

Diphenil exatrien probe (DPH), a lipid-like molecule, was added to the lipidic mixture dissolved in chloroform/methanol 9:1 in a 1:1000 ratio with respect to lipid from an ethanol stock; the LUVs were then prepared as described above. It has been proven that DPH has a negligible effect on bilayer fluidity at this lipid/probe ratio [73]. DPH fluorescence anisotropy was then recorded upon the addition of 1 to 12 µM Trematocine to a 200 µM lipid suspensions of LUVs of different compositions (100% PC, 70:30 PC/PG, and 3:1 PE/PG), with 5 nm emission and excitation bandwidths and 360 nm/450 nm excitation/emission wavelengths, respectively. Anisotropy (r) values were normalized using r_0_ LUVs values, i.e., in the absence of Trematocine.

### 4.7. Antimicrobial Activity of Trematocine

The antimicrobial activity of Trematocine peptide was examined against the yeast *Candida boidinii*, the Gram-positive bacteria *Bacillus pumilus*, and two Gram-negative bacteria, *Escherichia coli* and *Psychrobacter* sp. TAD1. MIC was determined by the broth microdilution method in 96 well polystyrene microplates Greiner Bio-One™ (Sigma Darmstadt, Germany) and streptomycin sulfate was used as the positive control. Besides *Psychrobacter* sp. TAD1, which was grown at 15 °C, all the other strains were grown aerobically and cultured at 28 °C. The growth medium without peptide was used as the negative control. The peptide was diluted (0.5–100 μM) in Mueller Hinton Broth, and 100 µL of each dilution was dispensed into each well. The inoculum suspension was adjusted to achieve 5 × 10^6^ CFU mL^−1^. The MIC was defined as the lowest concentration of the peptide that totally inhibited the growth. An aliquot (5 µL) of the cell suspension was taken from the above MIC microwell plate, and the cell suspension was plated on an LB agar Potato Dextrose Agar (PDA), for determining Minimal bactericidal Concentration (MBC) and Minimum Fungicidal Concentration (MFC), respectively. The MBC and MBT were defined as the lowest peptide concentrations at which more than 99.9% of the cells were killed compared with an untreated control.

### 4.8. Haemolytic Activity Assay 

The haemolytic assay was performed against rabbit erythrocytes maintained in Alsever’s solution (Innovative Research). Before the assay, the Alsever’s solution was removed and erythrocytes were resuspended in PBS 1X. Erythrocytes were successively counted, and a suspension of 5,000,000 red blood cells was incubated with serial dilutions (from 5 µM to 200 µM, six dilutions) of Trematocine. As a negative control, we used erythrocytes in PBS, while as a positive control we used erythrocytes in triton 10% *v*/*v*. The plate was incubated at 37 °C for 2 h and subsequently centrifuged at 1200 rpm × 3 min to separate the pellet from the supernatant. Each point was made in triplicate. The absorbance was measured at 570 nm. The relative OD compared to the positive control defined the percentage of haemolysis [74].

### 4.9. Cytotoxicity Assay 

The cytotoxicity of the peptide was tested on primary human fibroblast cell lines (FB789) grown in Dulbecco’s Modified Eagle Medium (DMEM). The cytotoxicity of Trematocine was determined by measuring the intracellular adenosine triphosphate (ATP) levels using the luciferase-based ATPlite assay (PerkinElmer, Waltham, MA, USA), according to the maufacturer’s instructions. Cells were seeded on 96-well microplates at a concentration of 5 × 10^3^ cells per well in 100 μL of medium for 8 h and 24 h at 37 °C in a humidified incubator with 5% CO_2_ atmosphere. Serial dilutions of peptide solutions (from 3.12 µM to 100 µM, six dilutions) dissolved in water were added; as a negative control the ATP level in cells grown in normal medium without the peptide was used, while as a positive control, cells added with NaN3 10% *v*/*v* were used. After 8 and 24 h, the cells were lysed, and the lysates were transferred into opaque well plates (Optiplate-96, PerkinElmer). Emitted light amount, linearly correlated with ATP concentration, was measured with a microplate luminometer (Victor II PerkinElmer) for 10 min in the dark. Three replicates for each dilution were performed. Cell viability values were expressed as the mean + SD and calculated as the percent values of the treated samples with respect to the untreated cells. The differences from the control were considered significant if *p* < 0.05, using two-way ANOVA analysis followed by the Bonferroni’s post-test.

## 5. Conclusions

In conclusion, we have identified a new antimicrobial peptide from a very peculiar source; a fish from Antarctica. We evidenced that it interacts with and creates pores on bacteria cell membranes and we determined its high antimicrobial activity against some model Gram-negative bacteria. Moreover, we demonstrated that it exerts no toxicity against both erythrocytes and primary mammalian cell lines. This new antibiotic molecule will be successively tested against drug-resistant human pathogens to verify the possible use as an alternative antimicrobial agent in conjunction with studies related to the analysis of its pharmacokinetics and pharmacodynamics properties.

## Figures and Tables

**Table 1:** Partition constant values relative to the different LUV compositions.

LUV Composition	*K_x_* (1 × 10^4^)	r^2^
PC/PG (70:30)	8.84 ± 1.5	0.99
100% PC	4.7 ± 0.6	0.99
PE/PG (3:1)	4.2 ± 0.3	0.99

**Table 2:** Stern–Volmer constant relative to Trematocine in the presence/absence of LUVs of different compositions. Net Accessibility Factor (NAF) is defined as in Equation (2).

LUV Composition	*K_SV_*	NAF_(1)_
PC/PG (70:30)	1.33 ± 0.05	0.063
100% PC	1.31 ± 0.03	0.062
PE/PG (3:1)	0.94 ± 0.06	0.044
Buffer	21.16 ± 0.08	1

**Table 3:** Antimicrobial activity of Trematocine.

Microrganism	MIC	MBC/MFC
	**Trematocine (μM)**	
*Escherichia coli*	25	25
*Bacillus pumilus*	10	25
*Psychrobacter* sp. (TAD1)	2.5	10
*Candida boidinii*	50	100

**Table 4:** Primers used for cloning and expression analysis.

Gene	Primers Sequence 5′-3′ (Forward, FW, and Reverse, RV)	Accession Number
β-actin	ATGTACGTTGCCATCC (FW) GAGATGCCACGCTCTC (RV)	AJ493428
Trematocine	GTGCACCATCGTCTTTCTGGTGC (FW, complete sequence) GGCAAACGCTCGCTCTCGGTC (RV, complete sequence)	MH325166
Trematocine	GTGCACCATCGTCTTTCTGGTGC (FW, real-time) GGCAAACGCTCGCTCTCGGTC (RV, real-time)	MH325166
18 S ribosomal RNA	CCAACGAGCTGCTGACC (FW, real-time PCR) CCGTTACCCGTGGTCC (RV, real-time PCR)	AY831388

## Abbreviations

AMP	antimicrobial peptide
DPH	1,6-diphenil-exatrien
OM	outer membrane
IM	inner membrane
PC	1-palmitoyl-2-oleoyl-glycero-3-phosphocholine
PG	1-palmitoyl-2-oleoyl-sn-glycero-3-phosphoglycerol
PE	1-palmitoyl-2-oleoyl-sn-glycero-3-phosphoethanolamine
MIC	minimum inhibitory concentration
MBC	minimum bactericidal concentration
MFC	minimum fungicidal concentration
CFU	colony forming units
LUV	large unilamellar vesicle
DEPC	diethyl pyrocarbonate
ROX	6-carboxy-X-rhodamine
EDTA	ethylenediamin tetraacetic acid
PBS	phosphate buffered saline
OD	optical density
ANS	1-aminonaphtalene-8-sulfonic acid
